# Evaluation of Gum of *Moringa oleifera* as a Binder and Release Retardant in Tablet Formulation

**DOI:** 10.4103/0250-474X.45400

**Published:** 2008

**Authors:** D. S. Panda, N. S. K. Choudhury, M. Yedukondalu, S. Si, R. Gupta

**Affiliations:** Institute of Pharmacy and Technology, Salipur, Cuttack-754 202, India; 1 Department of Pharmacy, SCB Medical College Cuttack-754 202, India; 2 College of Pharmaceutical Sciences, Mohuda, Berhampur-760 001, India; 3 School of Pharmacy, ITER, Bhubaneswar-751 030, India; 4 Birla Institute of Technology, Mesra, Ranchi-835 215, India

**Keywords:** Binder, gum, *Moringa oleifera*, release retardant, tablet

## Abstract

The present study was undertaken to find out the potential of gum from *Moringa oleifera* to act as a binder and release retardant in tablet formulations. The effect of calcium sulphate dihydrate (water insoluble) and lactose (water soluble) diluent on the release of propranolol hydrochloride was studied. The DSC thermograms of drug, gum and mixture of gum/drug indicated no chemical interaction. Tablets (F1, F2, F3, and F4) were prepared containing calcium sulphate dihydrate as diluent, propranolol hydrochloride as model drug using 10%, 8%, 6% and 4% w/v of gum solution as binder. Magnesium stearate was used as lubricant. Physical and technological properties of granules and tablets like flow rate, Carr index, Hausner ratio, angle of repose, hardness, friability and disintegration time were determined and found to be satisfactory. Tablets were prepared by wet granulation method containing calcium sulphate dihydrate as excipient, propranolol hydrochloride as model drug using 10%, 20% and 30% of gum as release retardant, magnesium stearate was used as lubricant. Similarly tablets were prepared replacing lactose with calcium sulphate dihydrate. Despite of the widely varying physico-chemical characteristics of the excipients, the drug release profiles were found to be similar. The drug release increased with increasing proportions of the excipient and decreased proportion of the gum irrespective of the solubility characteristics of the excipient. The values of release exponent ‘n’ are between 0.37 and 0.54. This implies that the release mechanism is Fickian. There is no evidence that the dissolution or erosion of the excipient has got any effect on the release of the drug. The t_50%_ values for tablets containing calcium sulphate dihydrate were on an average 10%-15% longer than the tablets containing lactose as excipient. These relatively small differences in t_50%_ values suggest that the nature of excipient used appeared to play a minor role in regulating the release, while the gum content was a major factor.

Additives play an important role in pharmaceutical preparations like tablet, lotions, suspensions, syrups and ointments. Recent trends towards the use of the vegetable and nontoxic products demand the replacement of synthetic excipients with natural ones. Vegetable gums provide appropriate solution to the current problem. There are several reports about the successful use of hydrophilic polymers derived from plant, like guar, carrageenan, karaya, locust bean gum in pharmaceutical preparations^1^. Gum karaya has been used as carrier for the dissolution enhancement of a poorly water soluble drug nimodipine^2^. *Abelmoschus esculentus* gum has been used as mini matrix for furosemide and diclofenac sodium tablets^3^. Cashew-tree exudates have been used as a novel bioligand tool^4^. Seed gum of *Cassia tora* has been evaluated as a binder in tablets^5^. *Plantago ovata* and *Trigonella foenum graecum* mucilages have been evaluated for its binding properties^6^. Guar gum has been investigated for its application in colon specific dosage forms^7^. Gum of the tree *Moringa oleifera* been reported to have gel forming potential for topical application^8^.

In view of the easy availability of the plant, the exudate from the stem of the tree *Moringa oleifera* was investigated for its application as a binder and release retardant in tablet formulation. Propranolol hydrochloride was used as a model drug. *Moringa oleifera* is a small genus of quick growing tree distributed in India. The stem of the tree exudes a gum which is initially white in colour but changes to reddish brown to brownish black on exposure. It is sparingly soluble in water but swells in contact with water giving a highly viscous solution. It is polyuronide consisting of arabinose, galactose and glucoronic acid in the proportion of 10:7:2, rhamnose is present in traces^9^.

## MATERIALS AND METHODS

Propranolol hydrochloride was obtained from Ajanta Pharma (Mumbai, India), Gum of moringa was isolated in the laboratory as per previously reported procedure^8^. Calcium sulphate dihydrate, lactose and magnesium stearate were purchased from SD fine Chemicals Ltd (Mumbai, India), All other materials used were of analytical grade.

### Selection of excipients:

Lactose (freely water soluble), calcium sulphate dihydrate (water insoluble) are commonly used as diluents in tablet formulations. Having opposite solubility characteristics, it is reasonable to expect that lactose and calcium sulphate dihydrate would exhibit significant differences in drug release from hydrated gum matrices. It has been reported that lactose produced increased release rates of various drugs from polymeric matrices^10^,^11^. Lactose diffuses outwards through the gel layer increasing the porosity and decreasing the tortuosity of the diffusion path of drug, on the other hand calcium sulphate dihydrate may form porous, insoluble and non soluble matrix which may be of use in controlling the release of water soluble drug as the report about the use of calcium phosphate dihydrate^12^. In the present work, various concentration of gum solution was used as binder in preparing the tablets and higher proportions of gum was used as release retardant in the matrix tablet. Calcium sulphate dihydrate and lactose were used separately as diluents, to find out their effect on the release behavior of the drug.

### Differential scanning calorimetry:

The DSC curves of gum, propranolol hydrochloride and mixture of the gum/propranolol hydrochloride were generated by a differential scanning calorimeter (DSC 220C, SEIKO, Japan) at heating rate of 10°/min from 60 to 280°.

### Preparation of the granules:

All the materials were passed through a mesh sieve with aperture of 250 μm before use. The tablets were prepared by wet granulation method. The compositions of the tablets are given in Tables 1 and 2. All the materials except magnesium stearate were thoroughly mixed in a tumbling mixer for 5 min and the solution of the gum of specified concentration was prepared by dispersing the gum in water. The gum solutions were used for moistening the powder mixture in mortar for preparing tablets to evaluate the binding potential, where as the tablets where gum was used as release retardant the powder mixture was moistened with water. The wet mass was then passed through a 500 μm mesh sieve and dried at a temperature not exceeding 60° for 1h. The dried granules were rescreened through a 300 μm sieve. The granules were evaluated for their flow properties, the flow rate through a funnel, the Carr index (compressibility index) and Hausner ratio was determined. Using the glass funnel specified in the European Pharmacopoeia-III the flow rate (g/s) was calculated from the time needed for the entire sample (40 g) to empty from the funnel. Bulk density was calculated from the amount of granules poured into a 100 ml graduated cylinder up to a total volume of 50 ml while for the tap density determination the cylinder was tapped until no measurable change in the volume was observed. Based on bulk and tap density both the Carr Index (%) [(tapped-bulk)×100/tapped] and Hausner ratio (tapped/bulk) were calculated. Angle of repose was determined by fixed funnel method^13^,^14^. Funnel with the end of the stem cut perpendicular to the axis of symmetry was secured with its tip at a given height (H) above a graph paper placed on a flat horizontal surface. The material was carefully poured through the funnel until the apex of the conical pile so formed just touches the tip of the funnel. The mean diameter (2R) of the base of the powder cone was determined and the tangent of the angle of repose is given by tanα = H/R, where α is the angle of repose.

**TABLE 1:** COMPOSITION OF TABLETS CONTAINING THE GUM AS BINDER

Ingredient	F1	F2	F3	F4
Propranolol HCl	40 mg	40 mg	40 mg	40 mg
Calcium sulphate dihydrate q.s	250 mg	250 mg	250 mg	250 mg
Gum solution (2ml)	10% w/v	08% w/v	06% w/v	04% w/v
Magnesium stearate	1%	1%	1%	1%

q.s. denotes quantity sufficient

**TABLE 2:** COMPOSITION OF TABLETS CONTAINING THE GUM AS RELEASE RETARDANT

Ingredient	F5	F6	F7	F8	F9	F10
Propranolol HCl	40 mg	40 mg	40 mg	40 mg	40 mg	40 mg
Calcium sulphate dihydrate q.s.	250 mg	250 mg	250 mg	-	-	-
Lactose q.s.	-	-	-	250 mg	250 mg	250 mg
Gum solution (2 ml)	10% w/w	20% w/w	30% w/w	10% w/w	20% w/w	30% w/w
Magnesium stearate	1%	1%	1%	1%	1%	1%

q.s. denotes quantity sufficient

### Production of tablets:

The granules were lubricated with 1% w/w magnesium stearate and compressed to tablets of diameter of 5.5 mm weighing 250 mg using (Cadmach Machinery Co. Pvt. Ltd. India) tablet punching machine.

### Tablet properties:

The tablets were evaluated for hardness using Monsanto hardness tester. The hardness reported is an average of three measurements. Twenty numbers of tablets were weighed and placed in a (Roche friabilator). After rotating for 4 min that is 100 revolutions. The percentage loss of weight was determined as an indicator of friability. The disintegration test was performed in water at 37° using an Erweka GmbH apparatus (Type 712, Erweka, Offenbach, Germany). The disintegration times reported are average of three determinations.

### *In vitro* dissolution study:

*In vitro* dissolution study was carried out on tablets, where gum has been used as release retardant. The USP XXII paddle method (Electrolab) was used with a constant temperature water bath at 37±0.5°. The dissolution media used was phosphate buffer pH 7.2. The speed of rotation was 100±1 rev/min. The samples were withdrawn in every 60 min over a period of 12 h. Triplicate studies were performed, the cumulative percentage of D calculated (±SD) and plotted against time.

### Analysis of propranolol hydrochloride in matrix tablets and dissolution medium:

Stock solution (100 μg/ml) of the propranolol hydrochloride in phosphate buffer was prepared. Diluting the stock solution with the phosphate buffer pH 7.2 gradient solutions of concentration 10, 20, 30, 40, 50 and 60 μg/ml were prepared and their absorbances were taken (UV/Vis spectrophotometer, Shimadzu, Japan) at 290 nm. The calibration curve was prepared by plotting the absorbance against concentration. This calibration curve was used for the quantitative determination of propranolol hydrochloride in matrix tablets and dissolution medium.

### Data analysis:

Korsemeyer *et al*. derived a simple relationship (1) which describes drug release from polymeric system^15^, *M_t_/M_∞_ = k.t^n^* (1), where M_t_/M_∞_ is the fraction of drug released, *t* is the release time, *k* is a constant incorporating structural and geometric characteristics of the release device and *n* is the release exponent indicative of the mechanism of release. Values for *n* and *k* for each matrix formulation were obtained by plotting the logarithm of the fractional release against the logarithm of time. The slope of the line is *n* while log *k* is the intercept. The drug release data were plotted using values of M_t_/M_∞_ within the range of 0.10-0.60 and the values of *n* and *k* calculated by regression analysis (values of 95% confidence limit). The *n* and *k* values were calculated from plots of M_t_/M_∞_ within the range of 0.10-0.60 because, in the initial stages (<0.10), the drug release rate is usually very rapid and above 0.60 it tends to slow down with time.

### Statistical analysis:

The cumulative percent release of propranolol hydrochloride from tablets containing calcium sulphate dihydrate and lactose was compared and the statistical significance was tested using student's t-test. A value of *P< 0.05* was considered statistically significant.

## RESULT AND DISCUSSION

**Fig. 1:**
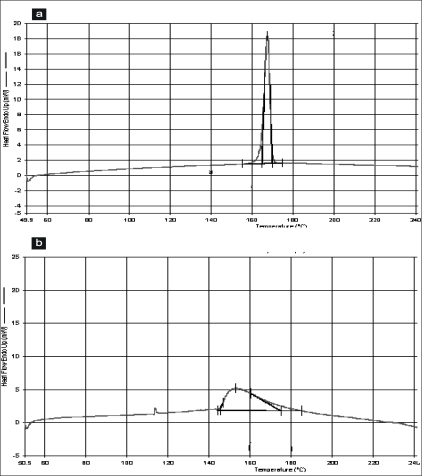
DSC thermogram of propranolol hydrochloride and gum DSC thermogram of a. propranolol hydrochloride and b. gum

**Fig. 2:**
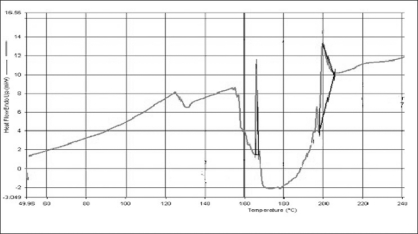
DSC thermogram of mixture of propranolol hydrochloride and gum

DSC thermograms of propranolol hydrochloride, gum and mixture are depicted in figs. 1a,b and 2, respectively. The thermogram of the pure drug exhibited a sharp endothermic peak at 167.51° corresponding to its melting point, while the gum exhibited a broad endothermic peak at 153.02° owing to its amorphous nature. The DSC thermogram of the gum and drug mixture showed identical peaks corresponding to pure drug indicated the absence of well defined chemical interaction between the drug and the gum.

Flow properties of the granules were determined as good flowability is prerequisite for the preparation of the tablets with an acceptable weight variation. For all the formulations the flow rate of the granules were between 6 and 9 g/s. according to literature data excellent flow properties are seen for powders with a Carr Index between 5 and 15% and a Hausner ratio below 1.25^16^. All the formulations tested had a Carr Index ranging between 7.1 and 12.6% while their Hausner ratio was below 1.25. The angle of repose was found to be between 17 and 22. The excellent flow properties were also proved by the narrow weight distribution of the tablets, as all the formulations had coefficient of variation values of less than 6% relative to their mean weight. The hardness of the tablet varies between 6 and 8 kg clearly indicating that they are strong tablets and they can withstand the mechanical shocks. This is combined with the friability (less than 1%) of all the formulations demonstrated the effectiveness of the gum for use as binder. The disintegration time of the tablets varied between 4 and 6 min irrespective of the hardness, the swelling property of the gum might be attributing to the faster disintegration of the tablets (Table 3).

**TABLE 3:** PHYSICAL AND TECHNOLOGICAL PROPERTIES OF THE GRANULES AND TABLETS

Properties	F1	F2	F3	F4
Flow rate(g/s)	7.3	6.9	6.2	8.9
Carr index (%)	11.2	7.5	12.4	7.1
Hausner ratio	1.03	1.12	1.09	1.07
Angle of repose	17	18	22	21
Hardness (kg)	7	7	8	7
Friability (%)	0.6	0.6	0.3	0.2
Disintegration Time	4.6	4.4	5.8	5.8

Results are mean of three observations

**Fig. 3:**
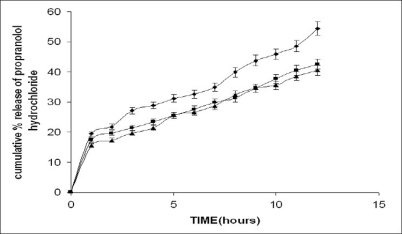
*In vitro* release profile of drug from tablets containing calcium sulphate dihydrate. F5 containing 10% gum (–◆–), F6 containing 20% gum (–■–) and F7 containing 30% gum (–▲–) (n=3).

**Fig. 4:**
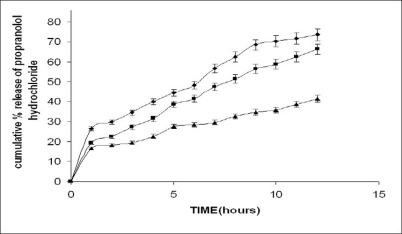
*In vitro* release profile of drug from tablets containing calcium lactose. F8 containing 10% gum (–◆–), F9 containing 20% gum (–■–) and F10 containing 30% gum (–▲–) (n=3).

The present work involves the evaluation of the gum from *Moringa oleifera* for its ability to retard the release of propranolol hydrochloride from tablets and the effect of excipients of opposite solubility on the release of the drug. The dissolution study was carried out on formulations F5-F10 (Table 2). Despite of the widely varying physico-chemical characteristics of the excipients, the drug release profiles were found to be similar. The drug release increased with increasing proportions of the excipient and decreased proportion of the gum irrespective of the solubility characteristics of the excipient the typical release profile of the tablets containing calcium sulphate dihydrate and lactose are shown in figs. 3 and 4. The release exponent *n* and R^2^ values for the formulations are given in the Table 4. The tabulated data shows that values of n are between 0.37 and 0.54. This implies that the release mechanism is Fickian. Much variation was not observed in the n value. There is no evidence that the dissolution or erosion of the excipient has got any effect on the release of the drug. The t_50%_ values for tablets containing calcium sulphate dihydrate were on an average 10%-15% longer than the tablets containing lactose as excipient. These relatively small differences in t_50%_ values suggest that the nature of excipient used appeared to play a minor role in regulating the release, while the gum content was a major factor. Lower gum content would result reduced swelling with corresponding decrease in diffusional path length. Moreover the excipient would either enhance dissolution or erosion mechanism, depending on the solubility of the excipient, which compensates for the slowing diffusion rate through the gradually increasing gel layer by creating greater porosity for the drug pathway.

**TABLE 4:** RELEASE EXPONENT (N) AND R^2^ VALUES

Formulation	n	R^2^
F5	0.41±0.003	0.94±0.006
F6	0.37±0.007	0.93±0.008
F7	0.41±0.002	0.95±0.012
F8	0.47±0.006	0.95±0.002
F9	0.54±0.004	0.97±0.003
F10	0.39±0.005	0.94±0.003

R^2^ is the square of correlation coefficient; values are mean of three readings ± Standard deviation
